# Supplementary material for: Revision of Cytomegalovirus Immunoglobulin M Antibody Titer Cutoff in a Maternal Antibody Screening Program in Japan: A Cohort Comparison Involving a Total of 32,000 Pregnant Women
Source: Viruses. 2023 Apr 13;15(4):962. doi: 10.3390/v15040962 (PMC10143041; doi:10.3390/v15040962)
Supplement: Supplementary file 1 [file viruses-15-00962-s001.zip › viruses-2302053-supplementary.pdf]

Supplementary Materials

Congenital cytomegalovirus (CMV) infection cases in pregnant women with high IgG avidity (the original CMV IgM cutoff period)

Forty-eight pregnant women with positive/equivocal IgG, IgM under the revised cut-off, and high IgG avidity underwent neonatal urine CMV DNA tests. Two infants had congenital cytomegalovirus (cCMV) infection and were symptomatic. One showed fetal ascites, whereas the other showed a unilateral threshold elevation on auditory brainstem response tests (Cases 13 and 14 in main text Table 1).

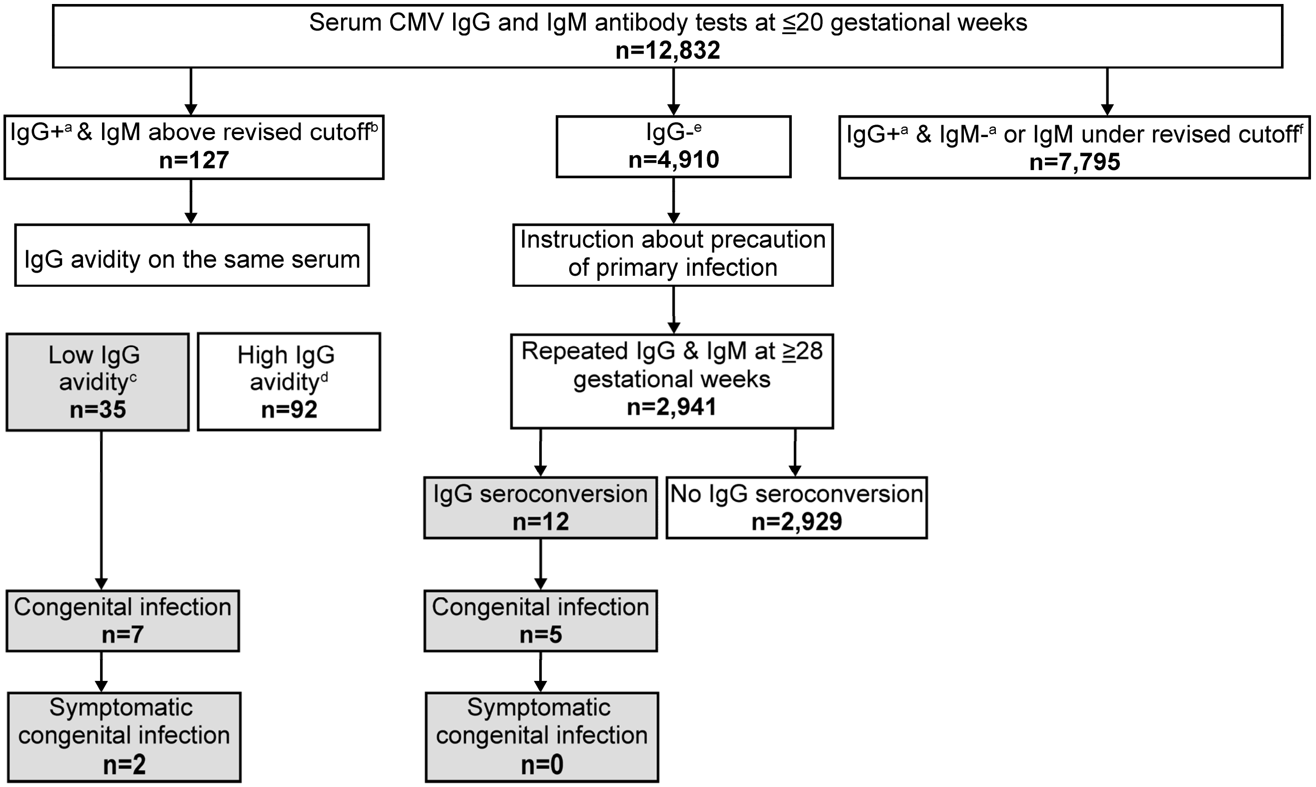

Supplementary Figure S1. Flowchart of the maternal cytomegalovirus (CMV) antibody screening program from April 2017 to March 2019, with the revised cutoff of CMV IgM antibody titer index. [a]including equivocal IgG. [b]≥4.00 IgM antibody titer index. [c]≤35.0% IgG antibody avidity index. [d]≥35.1% IgG antibody avidity index. [e]with any IgM antibody result. [f]<4.00 IgM antibody titer index.

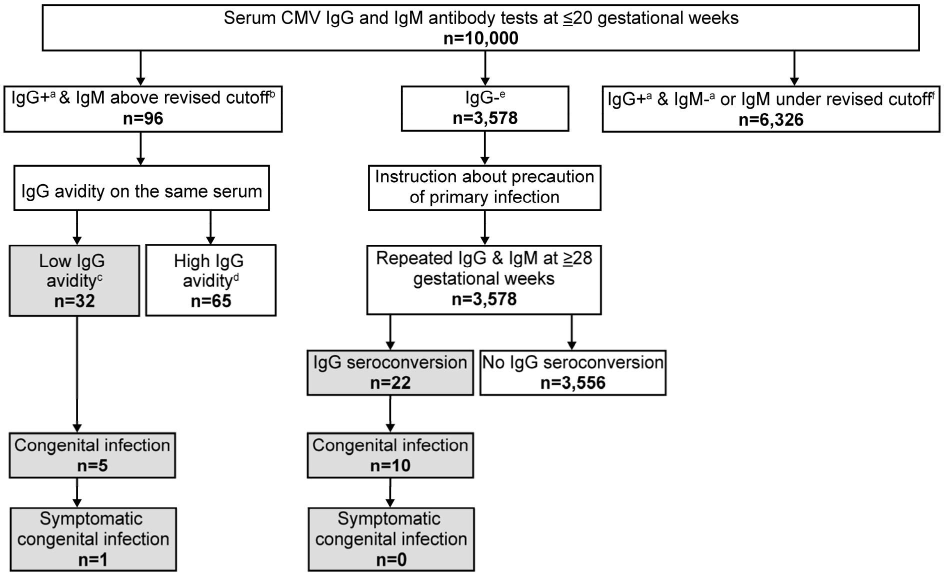

Supplementary Figure S2. Flowchart of the maternal cytomegalovirus (CMV) antibody screening program prior to 2017 converted by 10,000 pregnant women. [a]including equivocal IgG. [b]≥4.00 IgM antibody titer index. [c]≤35.0% IgG antibody avidity index. [d]≥35.1% IgG antibody avidity index. [e]with any IgM antibody result. [f]<4.00 IgM antibody titer index.

Supplementary Table S1. Proportions of pregnant women with low cytomegalovirus (CMV) immunoglobulin (Ig) G avidity and infants with congenital CMV infections among pregnant women with positive (including equivocal) IgG and IgM above the original cutoff (≥1.21) during the corresponding periods

Study period	April 2017–March 2019	September 2013–March 2017	P value
Cutoff of CMV IgM titer to measure IgG avidity as a reflex testing in maternal antibody screening program	The revised cutoff (≥4.00 index) of CMV IgM titer	The original cutoff (≥1.21 index) of CMV IgM titer	
Number of pregnant women with positive CMV IgG* & IgM above the original cutoff (≥1.21 index of IgM titer)	722	1,037	
Number of pregnant women with positive CMV IgG* & IgM above the revised cutoff (≥4.00 index of IgM titer)	127 (17.6%)	184 (17.7%)	0.95
Number of pregnant women with positive CMV IgG*, IgM above the revised cutoff, & low IgG avidity (≤35.0% of IgG avidity index)	35 (4.8%)	67 (6.5%)	0.18
Number of pregnant women with infant congenital CMV infection including symptomatic infection	7 (1.0%)	8 (0.8%)	0.79
Number of pregnant women with infant symptomatic congenital CMV infection	2 (0.3%)	1 (0.1%)	0.57

*Including equivocal IgG. CMV, cytomegalovirus; Ig, immunoglobulin

Copyright: © 2023 by the authors. Licensee MDPI, Basel, Switzerland. This article is an open access article distributed under the terms and conditions of the Creative Commons Attribution (CC BY) license (https://creativecommons.org/licenses/by/4.0/).
